# Editorial: Neurorepair Strategies to Induce Angiogenesis, Neurogenesis and Synaptic Plasticity

**DOI:** 10.3389/fcell.2021.740881

**Published:** 2021-08-12

**Authors:** Friederike Klempin, Fabricio Simão, Mauro Cunha Xavier Pinto

**Affiliations:** ^1^Department of Psychiatry and Psychotherapy, Charité – University Medicine Berlin, Berlin, Germany; ^2^Joslin Diabetes Center, Harvard Medical School, Boston, MA, United States; ^3^Department of Pharmacology, Institute of Biological Sciences, Federal University of Goiás, Goiânia, Brazil

**Keywords:** neurorepair, angiogenesis, gliogenesis, neurogenesis, synaptic plasticity

Brain damage as a result of ischemic stroke and trauma, following injury, or in neurodegenerative disease generates severe consequences leading to impairments in motor and cognitive functions. Although neuroplasticity persists in the adult human brain, the reorganization and self-repair are limited and recovery from most diseases is far from certain. Recent discoveries point to repair strategies of the adult injured or diseased human brain that require approaches targeting neuron replacement, angiogenesis, gliogenesis, and enhancing synaptic plasticity. To achieve an efficient regenerative response, the orchestration of scar formation, cleaning debris of damaged cells and integrating newborn neurons into existing circuitries is of importance. Knowledge of the various steps and the underlying neurochemical processes supporting cell genesis and integration, provides valuable insights of signaling pathway management that might lead to new therapeutic strategies in tissue repair.

For this special issue, we collected review articles and original research reports summarizing and discussing repair strategies for the rodent and human brain. The studies specifically focus on glial cells in the repair process, and examine the new exciting role of astrocytes (Chiareli et al.) and their reprograming potential following spinal cord injury (Puls et al.) or cortical brain damage (Ribeiro et al.). Thereby, two articles highlight the reprogramming capacity of reactive astrocytes *in vitro* that involves Galectin-3 triggering of Notch1 signaling in response to damage-facilitating nuclear translocation of Notch intracellular domain (Ribeiro et al.). Excitingly, Puls et al. reveal an *in vivo* astrocyte-to-neuron conversion, whereby reactive astrocytes covert into functional neurons *via* the NeuroD1 pathway that may improve neurorepair processes following spinal cord injury.

Besides astrocytes, gliogenesis of microglia and oligodendrocyte precursor cells (OPCs) in the adult brain promote repair processes and enhance signaling pathways that in turn contribute to neuroplasticity. Specifically, Neuron-Glia (NG) 2-positive OPCs constitute the main proliferating cell population outside the neurogenic niches, and display an endogenous source with multipotent potential. Importantly, demyelinating diseases require oligodendrocytes; and the review by Reyes-Haro et al. reveals OPCs express GABA_A_ receptors, communicate with neurons through GABAergic signaling, which might contribute to remyelination as repair strategy. In addition, the transcriptome profile of corpus callosum shows the activation of oligodendrogenesis alongside an angiogenic response in cerebral hypoperfused mice highlighting the role of glial cells as therapeutic target in vascular cognitive impairment and dementia (Takase et al.).

Microglia take part in neuroplasticity of the healthy rodent hippocampus and may be a potential therapeutic model in the maintenance of mood. In pathology, dysfunctional microglia contribute to late-stage development of Alzheimer's Disease (AD) inducing cell death and the release of brain-derived neurotrophic factor (Turkin et al.). Apolipoprotein E (APOE) is an important factor in the brain vasculature and also associated with an increased risk to develop AD by affecting cerebral vascular integrity, brain metabolism, synaptic plasticity, and neuroinflammation. In this issue, two studies present their findings and discuss the autocrine functions of *apoE* genotype in the modulation of basal phenotypic state of brain endothelial cells, the role of APOE4 in increased inflammation (Marottoli et al.), and in promoting tonic-clonic seizures (Lamoureux et al.).

The review by Cuartero et al. ask whether “Post-stroke Neurogenesis [is] Friend or Foe?” and provides an overview of the role of newborn neurons in the neurogenic niches subventricular zone and subgranular zone after ischemic stroke. The authors conclude that neurogenesis in the dentate gyrus may adopt a “maladaptive plasticity response” that contributes to “the development of post-stroke cognitive impairment and dementia.” Last but not least, Park and Hayakawa discuss the role of cell-free extracellular mitochondria in non-cell-autonomous signaling in central nervous system pathophysiology. Neurorepair strategies also include the use of (external) bioscaffolds and the creation of cell platforms providing the basis for tissue engineering (Zamproni et al.).

In conclusion, the present collection of articles reflects the complexity of the central nervous system and thus the varies repair strategies and mechanisms involved in the neurorepair process. Collectively, our studies provide an overview and explore different strategic approaches including gliogenesis, angiogenesis, neurogenesis, and targeting synaptic plasticity following distinct types of brain damage. Our approach in summarizing the specific findings in this edition is to encourage new young scientists to discover novel pathways, to inspire researchers to go new innovative ways—facilitating neurorepair process and leading to the development of novel solutions that improve the quality of life of patients following injury and in neurological disease.

## Author Contributions

FK, FS, and MP have made a substantial, direct and intellectual contribution to this editorial, and all authors have approved it for publication.

## Conflict of Interest

The authors declare that the research was conducted in the absence of any commercial or financial relationships that could be construed as a potential conflict of interest.

## Publisher's Note

All claims expressed in this article are solely those of the authors and do not necessarily represent those of their affiliated organizations, or those of the publisher, the editors and the reviewers. Any product that may be evaluated in this article, or claim that may be made by its manufacturer, is not guaranteed or endorsed by the publisher.

